# The Protective Effects and Immunological Responses Induced by a Carboxymethyl Cellulose Microcapsule-Coated Inactivated Vaccine Against Largemouth Bass Ranavirus (LMBRaV) in Largemouth Bass (*Micropterus salmoides*)

**DOI:** 10.3390/vaccines13030233

**Published:** 2025-02-25

**Authors:** Jiale Zhai, Yuding Fan, Yiqun Li, Mingyang Xue, Yan Meng, Zhenyu Huang, Jie Ma, Yong Zhou, Nan Jiang

**Affiliations:** 1 Division of Fish Disease, Yangtze River Fisheries Research Institute, Chinese Academy of Fishery Sciences, Wuhan 430223, China; 2 College of Fisheries, Huazhong Agricultural University, Wuhan 430070, China; 3 Department of Fish and Wildlife Science, University of Idaho, Moscow, ID 83844, USA

**Keywords:** *Micropterus salmoides*, largemouth bass ranavirus, vaccine, microcapsule

## Abstract

Background: Epizootics of largemouth bass ranavirus (LMBRaV) in largemouth bass (*Micropterus salmoides*) populations are associated with elevated mortality and significant financial losses. Given the lack of effective and safe medication to treat this disease, oral vaccination, which directly targets the intestinal mucosal immune system, is crucial for disease resistance. Methods: This study utilized carboxymethyl cellulose (CMC) to coat LMBRaV inactivated vaccine (LIV) (micro-CMC@LIV). The morphology and characteristics of the CMC microcapsules were determined. In vitro simulated gastric and intestinal conditions were used to validate that the microcapsules could tolerate gastric conditions and subsequently release their contents in the intestinal tract. This was confirmed using CMC-coated coumarin 6 (C6) fluorescence microcapsules. Results: After the oral administration of micro-CMC@LIV, the detection of LMBRaV major capsid protein confirmed effective antigen release and absorption in the midgut and hindgut. Neutralizing antibody titers were significantly higher (1:81.71) in the micro-CMC@LIV group compared to the uncoated vaccine group (1:21.69). The expression of genes linked to the innate and adaptive immune systems was upregulated post-micro-CMC@LIV treatment. Following the LMBRaV challenge, the micro-CMC@LIV group exhibited a relative percent survival (RPS) of 82.14%, significantly higher than the uncoated vaccine group (61.61%). Droplet digital PCR analysis revealed significantly lower viral loads in the liver, spleen, and head kidney of the micro-CMC@LIV group compared to the control group and the uncoated vaccine group. Conclusions: These results collectively suggest that the CMC-coated LIV can be effectively delivered to the intestinal tract and induce robust antibody and immune responses, providing a reliable method for preventing and controlling LMBRaV disease in the largemouth bass industry.

## 1. Introduction

The largemouth bass (*Micropterus salmoides*), a widespread freshwater fish native to North America, is now widely cultivated globally and is one of the most commercially traded fish species in China [1,2]. Since 2008, several outbreaks of largemouth bass ranavirus (LMBRaV) have occurred at *M. salmoides* farms in Foshan (Guangdong, China), subsequently spreading to Hubei [3,4,5]. LMBRaV belongs to the genus *Ranavirus* of the family *Iridoviridae* and is also known as Santee–Cooper ranavirus (SCRV) and largemouth bass virus (LMBV) [6,7]. LMBRaV infections result in high mortality rates, causing significant economic losses and threatening the global *M. salmoides* aquaculture industry [8]. According to the latest virus classification criteria published by the International Committee on Taxonomy of Viruses (ICTV), the *Iridoviridae* family can be divided into two subfamilies: *Alphairidovirinae* and *Betairidovirinae*. *Alphairidovirinae* comprises three genera, *Lymphocystivirus*, *Megalocytivirus*, and *Ranavirus*, primarily affecting vertebrates such as bony fish, amphibians, and reptiles. *Betairidovirinae* consists of three genera, *Iridovirus*, *Decapodiridovirus*, and *Chloriridovirus*, mainly infecting invertebrates like insects and crustaceans [9,10,11]. Despite the development and evaluation of various medicines and vaccines [4,8,12,13,14,15,16,17,18], further advancements in LMBRaV prevention are still needed.

Vaccination is widely recognized as one of the most effective methods for preventing diseases in large-scale commercial fish farms [19]. Vaccines can be categorized into live attenuated, inactivated, subunit, viral vector, and emerging non-viral vaccines, based on their production methods [20]. Currently, three primary vaccination methods are employed in fish: injection, immersion, and oral administration [21]. While injection is the most potent method, it is associated with higher processing costs and increased stress for the fish [22]. Immersion, another common method, is more practical but suffers from limited effectiveness due to poor antigen absorption across mucosal membranes [23]. Oral administration offers a more cost-effective and less stressful approach to vaccination [24]. It can trigger both systemic and mucosal immune responses by stimulating dendritic cells (DCs) [25,26]. Consequently, developing a successful oral vaccine to stop the development of fish infections is a promising strategy. Several LMBRaV yeast oral vaccines have been constructed, demonstrating relative percent survival (RPS) rates of 41.6% to 66.7% [15,17,18]. However, the development of even more effective oral vaccines remains an important area of research.

To develop an effective oral vaccine, a key challenge is to minimize antigen degradation in the gastrointestinal tract environment. Controlling antigen release and enhancing bioavailability through encapsulation is a promising strategy [27]. Microcapsules have demonstrated their effectiveness in vaccine delivery [28]. For instance, lecithin and carboxymethyl starch have been used to coat mesalamine, enabling controlled release and targeted delivery to specific areas of the gastrointestinal tract [29]. Carboxymethyl cellulose (CMC) is derived from cellulose, which is easily soluble in water [30]. Its biocompatibility, non-toxicity, renewability, and affordability have led to its widespread application in various biomedical fields, including drug delivery systems, wastewater treatment, and food packaging [31]. For example, CMC-coated gentamicin has been shown to form a sustainable hydrogel with controlled drug release [32]. Based on these properties, the CMC microcapsule-coated LMBRaV vaccine was selected for oral immunization of *M. salmoides*.

In this study, CMC microcapsules were used to coat LMBRaV inactivated vaccine (LIV), and their morphology and characteristics were determined. By detecting coated coumarin 6 (C6) and LMBRaV major capsid protein (MCP) in the intestinal tract, it was demonstrated that the CMC microcapsules could successfully deliver the vaccine to the hindgut. Moreover, the survival rate, serum neutralizing antibody titer, expression of immune-related genes, and viral copy number post-immunization were evaluated to analyze the protective efficacy and immunological responses induced by the CMC microcapsule-coated LMBRaV inactivated vaccine.

## 2. Materials and Methods

### 2.1. Preparation of Viral Stock, Cell Culture, and Experimental Fish

The LMBRaV-HB001 strain was stored in our laboratory (Luo et al., 2022 [3]). *Epithelioma papulosum cyprini* (EPC) cells were maintained at 25 °C in Medium 199 (M199, Hyclone, Logan, UT, USA) supplemented with 10% fetal bovine serum (FBS, Hyclone, Logan, UT, USA). Healthy *M. salmoides* (50 ± 5 g) were purchased from a farm in Wuhan City, Hubei Province, China, and were free of viruses, parasites, and bacteria. The fish were acclimated to a recirculating aquaculture system at 28 ± 1 °C for two weeks. *M. salmoides* were fed with commercial feed (≥48% protein, ≥6% fat, ≤12% water, and ≤12% ash, Tongwei, Chengdu, China) twice a day (at 9 a.m. and 6 p.m.), and the feeding amount was 2% of the body weight of the fish.

### 2.2. Preparation of LMBRaV Inactivation

LMBRaV (MOI = 0.1) was used to infect a monolayer of EPC cells. The virus was harvested when approximately 90% cytopathic effect (CPE) was observed. After three freeze–thaw cycles, the harvested virus was centrifuged at 5000× *g* for 10 min to discard the cell debris, and the viral supernatant was collected and stored at −80 °C. To determine the viral titer, the virus was serially diluted 10-fold in the M199 medium, and the 50% tissue culture infectious dose (TCID_50_/mL) was calculated using the Reed–Muench method [33]. LMBRaV was inactivated by treatment with 0.2% (*v*/*v*) formalin for 85 h at 37 °C.

The safety of the LMBRaV inactivated vaccine was evaluated through bacterial culture, cell culture, and in vivo studies, each experiment being repeated thrice. For the bacterial test, 100 μL of LIV was aseptically inoculated onto a BHI (Brain–Heart Infusion Broth) plate and incubated at 28 °C for 72 h, with no bacterial growth observed. In the cell culture test [34], EPC cells were inoculated with either 1 mL of LIV or LMBRaV and monitored for CPE for seven days. The viral supernatant, after three freeze–thaw cycles, was used to infect a new EPC monolayer, and this process was repeated twice. The absence of CPE in both rounds confirmed successful viral inactivation. For the in vivo test [18], ten healthy fish were orally administered with 2 mL of LIV. At 21 days post-immunization (dpi), samples from the foregut, midgut, and hindgut were collected for histological analysis.

### 2.3. Construction of Microcapsules

The LMBRaV inactivated vaccine was encapsulated in CMC and crosslinked with calcium using an emulsification/internal gelation technique adapted from Ghosh [35]. This technique was modified to enhance stability. To form the aqueous phase, 0.5 g CMC (Yuanye Bio-Technology Co., Ltd., Shanghai, China) was dissolved in 25 mL of double-distilled water (dd H_2_O), followed by the addition of 165 mg of CaCO_3_ and overnight stirring. The next day, 7.5 mL of LIV (1 × 10^7^ TCID_50_ mL^−1^) or 7.5 mL of PBS (control) was added and stirred for 30 min to ensure homogeneous dispersion within the CMC solution. For the oil phase, 1 mL of Span-80 was thoroughly dispersed in 19 mL of paraffin liquid (Macklin, Shanghai, China). The aqueous phase was then gradually introduced into the oil phase and stirred at 800 rpm for 15 min to form a water-in-oil emulsion. Subsequently, 10 mL of paraffin liquid containing 675 μL of glacial acetic acid was incorporated to initiate crosslinking of microcapsules. To break the emulsion, 100 mL of 0.85% NaCl solution with 2 mL of Tween-80 was added and stirred at 600 rpm for 10 min to harden the micro-CMC@LIV or micro-CMC. The microcapsules were collected by filtration and washed with ethanol and ddH_2_O. The morphology and characteristics of the microcapsules were then examined. Each experiment was repeated three times.

Microcapsule size: One hundred and fifty microcapsules were randomly selected from each independent experiment. The diameter of each microcapsule was measured using Cellsens Entry digital imaging software (Olympus 2.3). The mean diameter and standard deviation were calculated.

Loading efficiency: A high-concentration bacterial solution (OD_600_ > 2.0) was encapsulated in micro-CMC, and loading efficiency was assessed as previously described [36]. Encapsulation efficiency was determined by subtracting the OD_600_ of the micro-CMC in the micro-CMC@bacterial solution from the original OD_600_ of the bacterial solution. The loading efficiency of the microcapsules was calculated as follows:

$$Loading\;efficiency\left(\%\right)=\left(\frac{X_{1}-X_{2}}{X_{0}}\right)\times 100\%$$

where X_0_ is the OD_600_ of the initial bacterial solution before encapsulation, X_1_ is the OD_600_ of the final microcapsule solution coated with bacteria, and X_2_ is the OD_600_ of the control microcapsule.

Each 100 mL microcapsule solution contains 7.5 mL of LIV (1 × 10^7^ TCID_50_ mL^−1^). Based on the loading efficiency, the actual vaccine dose per milliliter of microcapsule was calculated as follows:

CMC vaccine titer (TCID_50_ mL^−1^) = 7.5 × 10^5^ × loading efficiency

Safety evaluation: A total of 15 healthy *M. salmoides* were orally administered with 2 mL of micro-CMC daily for three days. All fish were dissected seven days post-treatment, and their tissues were examined histopathologically.

### 2.4. Delivery Within the Gastrointestinal Tract

#### 2.4.1. Simulated Release In Vitro

The stability of micro-CMC under simulated fish gastrointestinal conditions was evaluated by incubating it in simulated gastric fluid (SGF, Leagene Biotechnology, Beijing, China) and simulated intestinal fluid (SIF, Leagene Biotechnology, Beijing, China) as described previously [36,37,38,39]. To assess gastric stability, 2 mL of micro-CMC was treated with 10 mL of SGF at 37 °C with oscillation. Morphological changes were observed and counted under a microscope at 1, 2, 3, 5, and 7 h post-treatment. For intestinal stability, micro-CMC was treated with SIF, and morphological changes were observed and counted at 30 min, 1, 5, 7, and 24 h post-treatment. Each experiment was repeated thrice. The release rate was calculated as follows:

$$Drug\;release\;\%=(1-\frac{\frac{\sum X_{i}}{n}}{\frac{\sum X_{0}}{n}})\times 100\%$$

where i is the sampling time point, n is the number of fields of view observed at random, X_0_ is the number of initial micro-CMC solutions, and X_i_ is the number of micro-CMC solutions at the sampling time point.

#### 2.4.2. C6 Fluorescence Verification

The hydrophobic fluorescent dye C6 was encapsulated within micro-CMC (micro-CMC@C6) to provide detectable fluorescence signals [40]. Ten fish were orally administered with 200 μL of micro-CMC@C6. At 3 and 6 h post-administration, three fish were euthanized at each time point. The complete intestinal tract was collected and dissected into foregut, midgut, and hindgut sections. The intestinal contents from the midgut and hindgut were collected and smeared onto slides. The presence of micro-CMC@C6 was observed under a fluorescence microscope. These experiments were repeated thrice.

#### 2.4.3. Detection of Micro-CMC@LIV in the Intestine

An immunofluorescence assay was employed to confirm the delivery of the vaccine to the midgut and hindgut of *M. salmoides* via micro-CMC@LIV. Ten fish were orally administered with 200 μL of micro-CMC@LIV. At 3, 6, and 9 h post-administration, three fish were euthanized, and their gut tissues were fixed in 4% paraformaldehyde. Frozen sections were prepared [18]. After washing and blocking, the midgut and hindgut sections were incubated with anti-LMBRaV MCP polyclonal antibody (1:500) at 4 °C, then incubated with Cy3-labeled goat anti-rabbit secondary antibody (1:1000, Beyotime, Shanghai, China). DAPI was used for nuclear staining. Finally, the sections were examined under a fluorescence microscope. These experiments were repeated thrice.

### 2.5. Oral Immunization and Sample Collections

**Figure 1:**
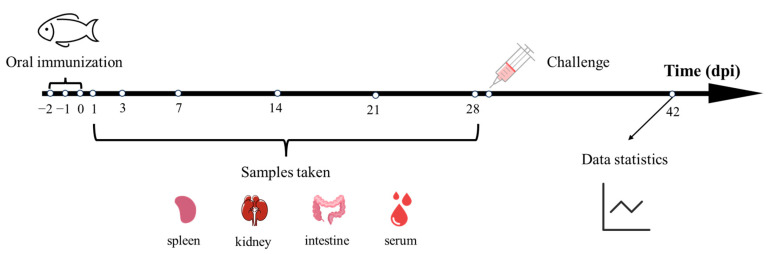
Schematic representation of the timeline for oral immunization and sample collection.

Four groups of healthy *M. salmoides* were randomly selected, each having three replicates with 65 fish per replicate. The micro-CMC@LIV group received 2 mL of micro-CMC@LIV (titer: 6.65 × 10^5^ TCID_50_ mL^−1^) orally. The micro-CMC group received 2 mL of micro-CMC orally. The LIV group received 2 mL of the same dose of inactivated vaccine without coating orally. The control group received 2 mL of PBS orally. After three days of continuous treatment, all fish were fed compound feed twice daily. The oral administration experiment lasted 28 days (Figure 1). At each sampling time point (1, 3, 7, 14, 21, and 28 days post-immunization (dpi)), nine *M. salmoides* from each group (three fish per replicate) were sampled. Blood samples were taken from each fish and stored at 4 °C for 2 h. After centrifugation for 5 min, the serum was separated and stored for subsequent analysis. Tissues (spleen, head kidney, and hindgut) were stored in TRIzol (Invitrogen, Carlsbad, CA, USA) at −80 °C for quantitative real-time PCR.

### 2.6. LMBRaV Challenge and Viral Load Analysis

At 28 dpi, four groups were established for a challenge test. Each group consisted of 40 *M. salmoides*. The above four groups were challenged by intraperitoneal injection of 100 μL of 1 × 10^7^ TCID_50_ mL^−1^ LMBRaV. Another group of 40 fish served as the control group and was challenged with 100 μL of PBS via intraperitoneal injection (named the PBS group). The water temperature was maintained at 28 ± 1 °C, and the fish were monitored for two weeks. The RPS of the vaccine was calculated as described previously [41]. Each experiment was repeated thrice. After the challenge, tissues (liver, head kidney, and spleen) were collected from three surviving fish in the micro-CMC@LIV group and the LIV group, and three surviving fish in the control group from each replicate for viral load determination using droplet digital PCR (ddPCR) [6]. Following weighing, the tissues were lysed, and viral DNA was extracted using the Viral DNA Extraction Kit (Omega Bio-tek, Norcross, GA, USA). Droplets were generated using the Bio-Rad QX200™ Droplet Generator (Bio-Rad, Hercules, CA, USA) and analyzed with the Bio-Rad QX200™ Droplet Reader (Bio-Rad, Hercules, CA, USA).

### 2.7. Serum Neutralization Antibody Assay

Serum neutralization antibody titers were determined according to a previous report [18]. Serum samples were heat-inactivated and serially diluted (1:2, 1:4, 1:8, 1:16, 1:32, 1:64, 1:128, 1:256, 1:512, and 1:1024) with M199 medium. In 96-well plates, 50 μL of each serum dilution was mixed with 50 μL of 10^2^ TCID_50_ mL^−1^ LMBRaV. Six duplicate wells were set up for each serum dilution, as well as positive and negative control wells. Two hours later, 100 μL of EPC cell suspension (10^6^ cells/well) was added to each well and incubated at 28 °C for 5 days. CPEs were observed and recorded daily. Antibody titers were determined using the Reed–Muench method [33]. These experiments were repeated thrice.

### 2.8. Expression Levels of Immune-Related Genes

Total RNA was extracted from the spleen, head kidney, and hindgut using TRIzol reagent (Invitrogen), followed by DNase treatment to remove genomic DNA [42]. cDNA was synthesized using the ReverTra Ace First Strand cDNA Synthesis Kit (Thermo Scientific, Waltham, MA, USA) and stored at −20 °C. The quantitative real-time PCR program was as follows: 95 °C for 10 min, followed by 40 cycles of 95 °C for 30 s and 60 °C for 30 s. Each reaction mixture contained 2 μL of diluted cDNA, 10 μL of Hieff qPCR SYBR Green Master Mix (Yeasen, Shanghai, China), 0.8 μL of forward and reverse primers, and 6.4 μL of sterile water. Gene expression levels were analyzed using the 2^−ΔΔCT^ method, which assumed 100% efficiency of qPCR assays [43,44], with *β-actin* as the internal control. The primer sequences used are listed in Table 1. These experiments were repeated thrice.

### 2.9. Data Analysis

The data are displayed as mean ± standard deviation (SD) values and were analyzed using one-way analysis of variance (ANOVA) in GraphPad software 8.0. A *p*-value ≤ 0.05 was considered statistically significant. The normal distribution of particle size was analyzed by the Shapiro–Wilk test.

## 3. Results

### 3.1. Morphological Characteristics of the Microcapsules

**Figure 2:**
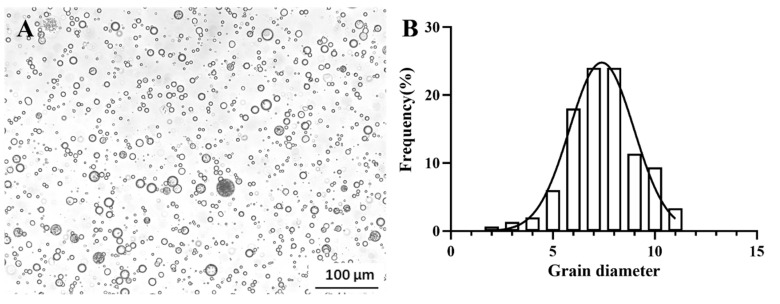
(**A**) Morphological characterization of the micro-CMC microcapsules. (**B**) The size distribution of the micro-CMC. Scale bar = 100 µm.

The micro-CMC exhibited a spherical shape (Figure 2A) with a mean particle size of 7.37 ± 0.47 μm (Figure 2B). By coating the bacterial solution, the mean loading efficiency of the micro-CMC was 88.67 ± 0.36%. The final concentration of the micro-CMC@LIV solution was 6.65 × 10^5^ TCID_50_ mL^−1^. The safety of the micro-CMC was assessed through histopathological examination of *M. salmoides* post-micro-CMC oral administration. No significant pathological changes were observed in the various tissues compared to the untreated control group (Supplementary Materials, Figure S1). The safety of LIV was also evaluated by cell culture; moreover, no significant differences in intestinal morphology were found between the LIV group and the control group (Supplementary Materials, Figures S2 and S3).

### 3.2. Micro-CMC Delivery Within the Gastrointestinal Tract

To confirm the delivery and release of micro-CMC@LIV in the gut, the in vitro stability of micro-CMC and the in vivo detection of micro-CMC@C6 and micro-CMC@LIV were investigated.

#### 3.2.1. Simulated Release In Vitro

**Figure 3:**
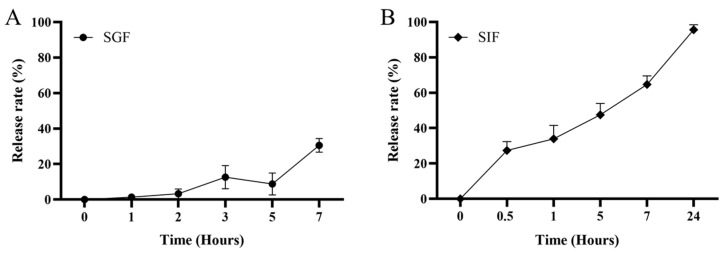
The release profile of microspheres under (**A**) SGF (pH = 1.5) and (**B**) SIF (pH = 6.8) conditions in vitro. The data are means for three replicates.

After treatment with SGF, micro-CMC was observed at 7 h, and its morphology remained stable. The mean release rate was 30.57 ± 3.84%. In contrast, after treatment with SIF, the number of micro-CMC particles decreased significantly over time. The mean release rate was 95.64 ± 1.73%. These results indicated that the release of micro-CMC primarily occurred in the gut (Figure 3).

#### 3.2.2. C6 Fluorescence Verification

**Figure 4:**
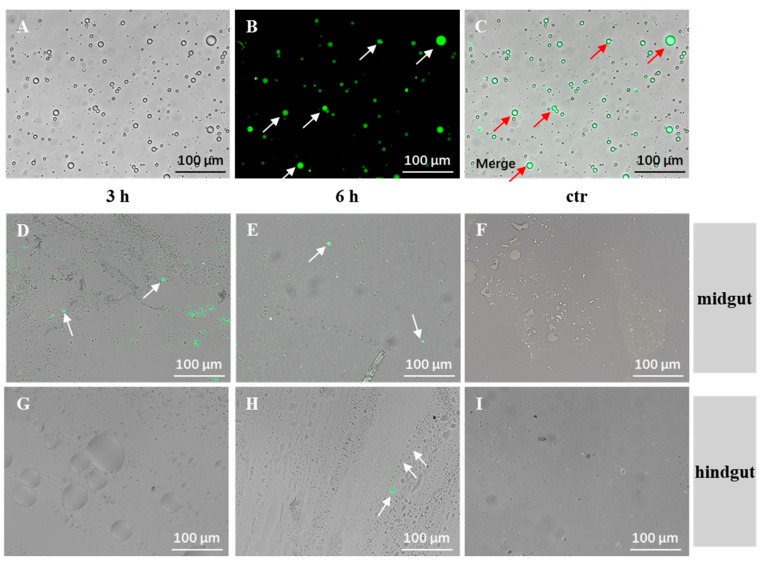
Micro-CMC@C6 distribution in the intestine following oral administration. (**A**–**C**) Fluorescent microspheres coated with C6. (**D**,**E**) Intestinal content smears from the midgut at 3 and 6 h post-administration. (**F**) Intestinal content smear from the midgut of untreated fish (control). (**G**,**H**) Intestinal content smears from the hindgut at 3 and 6 h post-administration. (**I**) Intestinal content smear from the hindgut of untreated fish (control). White arrows indicate fluorescent microspheres. Red arrows indicate the fluorescent microspheres in the same position after merge. Scale bar = 100 µm.

The prepared micro-CMC@C6 was used to visualize micro-CMC delivery to the gut under a fluorescent microscope. Following oral administration, fluorescent microcapsules were observed in the midgut at 3 and 6 h. In the hindgut, fluorescent microcapsules were observed at 6 h (Figure 4). These results indicate that micro-CMC@C6 successfully passed through the stomach and reached the midgut within 3 h and progressed to the hindgut after 6 h.

#### 3.2.3. Detection and Localization of Vaccine in the Intestine

**Figure 5:**
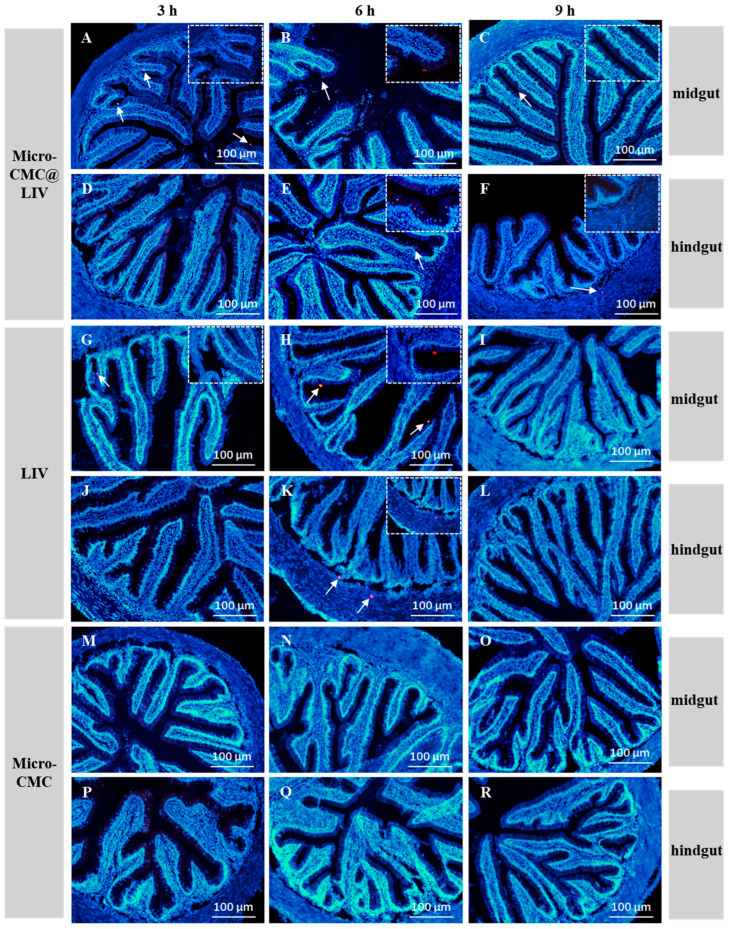
Immunofluorescence staining of the midgut and hindgut sections with the anti-MCP polyclonal antibody post-oral vaccination from 3 h to 9 h. (**A**–**C**) The midgut sections of the micro-CMC@LIV group sampled at 3, 6, and 9 h post-vaccination; (**D**–**F**) the hindgut sections of the micro-CMC@LIV group sampled at 3, 6, and 9 h post-vaccination; (**G**–**I**) the midgut sections of the LIV group sampled at 3, 6, and 9 h post-vaccination; (**J**–**L**) the hindgut sections of the LIV group sampled at 3, 6, and 9 h post-vaccination; (**M**–**O**) the midgut sections of micro-CMC group sampled at 3, 6, and 9 h post-treatment; and (**P**–**R**) the hindgut sections micro-CMC group sampled at 3, 6, and 9 h post-treatment. The anti-LMBRaV MCP polyclonal antibody was used as the primary antibody, and the nucleus was stained with DAPI. Arrows indicate the positive signals. The upper right corner is the detail map with a larger magnification. Scale bar = 100 µm.

To confirm the delivery efficacy, immunofluorescence staining using an anti-LMBRaV MCP antibody was performed. The results showed that fluorescence signals were detectable in the midgut of both the micro-CMC@LIV and LIV groups at 3 h post-vaccination (Figure 5A,G). No signals were observed in the hindgut at this time point (Figure 5D,J). At 6 h post-vaccination, fluorescence signals were evident in both the midgut and hindgut of both groups (Figure 5B,E,H,K). At 9 h post-vaccination, fluorescence signals were observed in both the midgut and hindgut of the micro-CMC@LIV group, while no signals were detected in either the midgut or hindgut of the LIV group (Figure 5C,F,I,K). All detected signals were localized to mucosal epithelial cells and submucosal cells (Figure 5A–C,E–H,K). No fluorescence signal was observed in the micro-CMC group (Figure 5M–R). Additionally, no signals were detected in the midgut and hindgut of the micro-CMC@LIV group at 6 h post-vaccination when the primary antibody was omitted (Supplementary Materials, Figure S4A,C). Similarly, no signals were detected in the midgut or hindgut of untreated fish (Supplementary Materials, Figure S4B,D). These findings indicate that micro-CMC@LIV effectively delivered the vaccine to the midgut and hindgut, where it was released. Moreover, the coated LIV appeared to persist in the gut for a longer duration compared to the uncoated LIV.

### 3.3. Oral Vaccine Protection Against LMBRaV

**Figure 6:**
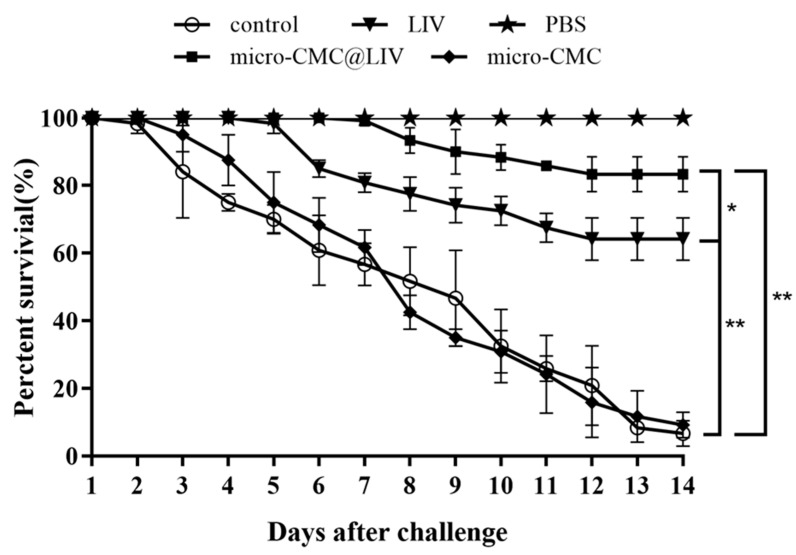
The survival rate of vaccinated and control largemouth bass after LMBRaV infection. Each group (n = 40) was intraperitoneally injected with PBS or LMBRaV (1 × 10^7^ TCID_50_ mL^−1^). The challenge was repeated three times. * *p* < 0.05; ** *p* < 0.01.

*M. salmoides* were infected with LMBRaV (1 × 10^7^ TCID_50_ mL^−1^) to evaluate the protective efficacy of the vaccine. The results of the challenge experiments are shown in Figure 6. Mortality in the control and micro-CMC groups began on the third day post-infection, peaking between days 4 and 7. At 14 days post-challenge, the mean survival rates of the control and micro-CMC groups were 6.67 ± 2.2% and 9.17 ± 2.2%, respectively. Mortality in the micro-CMC@LIV and LIV groups began to die at 7 and 5 days post-challenge, respectively, with mean final survival rates of 83.33 ± 3% and 64.17 ± 3.6%, respectively. The PBS group exhibited no mortality throughout the experimental period. The RPS values for the micro-CMC@LIV and LIV groups were 82.14% and 61.61%, respectively.

### 3.4. Determination of LMBRaV Viral Load After LMBRaV Challenge

**Figure 7:**
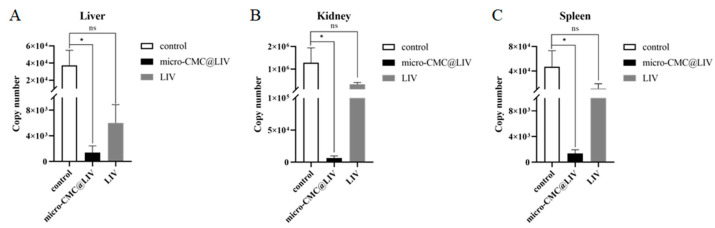
Detection of LMBRaV viral load in different tissues after LMBRaV challenge. (**A**) LMBRaV viral load in the liver; (**B**) LMBRaV viral load in the kidney; and (**C**) LMBRaV viral load in the spleen. * *p* < 0.05, ns = not detected.

To confirm whether oral administration of micro-CMC@LIV reduced viral load in immunized fish, the viral copy number in the liver, spleen, and head kidney of surviving fish from the micro-CMC@LIV group (n = 3), the LIV group (n = 3), and the control group (n = 3) was evaluated through ddPCR (Figure 7). The liver, spleen, and head kidney are the main target organs of LMBRaV infection in largemouth bass [46,47]. Significantly lower viral loads were detected in the liver, spleen, and head kidney of the micro-CMC@LIV group compared to the control group and the LIV group. Although the viral loads of each tissue in the LIV group were lower than in the control group, there is no obvious difference.

### 3.5. Serum Antibody Levels

**Figure 8:**
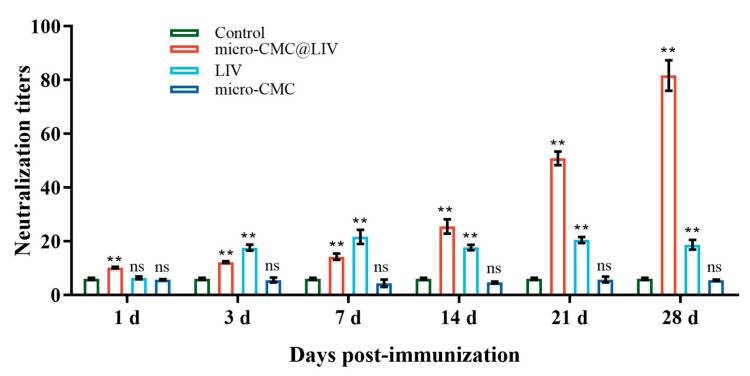
Determination of serum neutralizing antibody titers in largemouth bass following vaccination (1 to 28 days post-vaccination). Neutralization titers were determined as the reciprocal of the highest serum dilution that completely inhibited CPE in EPC cells inoculated with LMBRaV. The data are means for three replicates (3 fish/replicate) and presented as the means ± SD. ** *p* < 0.01; ns = not detected.

The serum neutralization titer was employed to measure the vaccine-induced target immune reaction. In the micro-CMC@LIV group, serum antibody levels steadily increased from 1 dpi to a peak level of 1:81.71 at 28 dpi. In the LIV group, antibody titers increased at 3 dpi and reached a peak level of 1:21.69 at 7 dpi. No significant increase in antibody titer was observed in the micro-CMC group (Figure 8).

### 3.6. Expression of Immune-Related Genes

**Figure 9:**
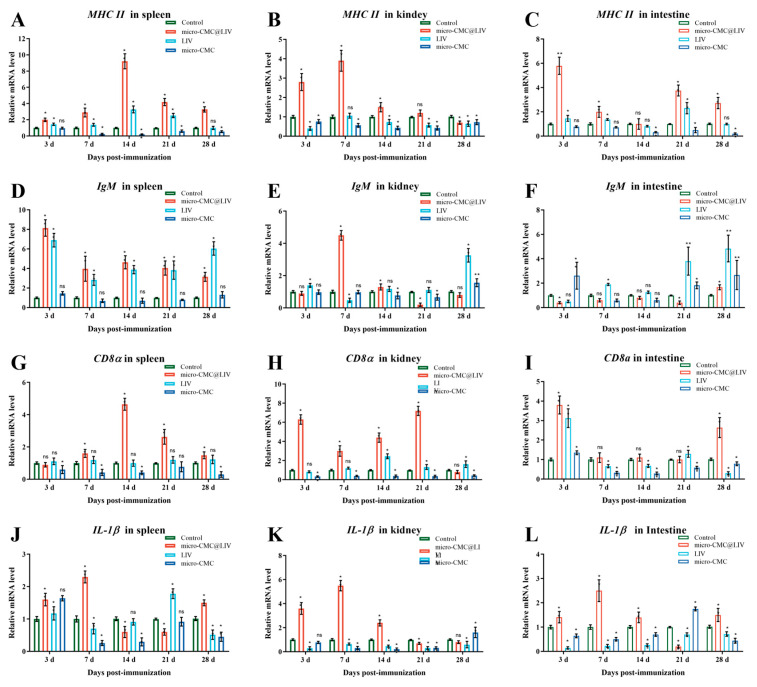
qRT-PCR analysis of the expression levels of immune-related genes of the spleen, head kidney, and hindgut post-vaccination. (**A**–**C**): Expressions of *MHCⅡ* in the spleen (**A**), kidney (**B**), intestine (**C**). (**D**–**F**): Expressions of *IgM* in the spleen (**D**), kidney (**E**), intestine (**F**). (**G**–**I**): Expressions of *CD8*α in the spleen (**G**), kidney (**H**), intestine (**I**). (**J**–**L**): Expressions of *IL-1β* in the spleen (**J**), kidney (**K**), intestine (**L**). The mRNA level of each gene was normalized in reference to the expression of the *β-actin* gene. For each gene, the mRNA level of the control animals was set as 1. The data are presented as mean S.D. (n = 3). * *p* < 0.05; ** *p* < 0.01, ns = not detected.

Immune responses following immunization were assessed by examining the expression levels of immune-related genes in the spleen, head kidney, and hindgut. The hindgut of fish is considered the primary site for antigen absorption and has a vital function in immune responses [48,49]; the spleen and kidney are the well-known important immune tissues in fish [46]. The transcription levels of *mhc II* in the spleen, head kidney, and hindgut of the micro-CMC@LIV group peaked at 14 dpi, 7 dpi, and 3 dpi, respectively, and subsequently declined (Figure 9A–C). In the LIV group, *mhc II* transcription was upregulated in the spleen and hindgut but downregulated in the head kidney (Figure 9A–C). The highest expression levels in all three tissues were observed in the micro-CMC@LIV group.

In addition, *igM* transcription in the spleen and kidney of the micro-CMC@LIV group peaked at 3 dpi and 7 dpi, respectively, and subsequently declined (Figure 9D,E). In the hindgut, *igM* transcription did not increase until 28 dpi (Figure 9F). In the LIV group, *igM* transcription was upregulated in the spleen from 3 dpi to 28 dpi (Figure 9D). In the head kidney and hindgut, transcription levels were significantly increased at 28 dpi and 21 dpi, respectively (Figure 9E,F). The *igM* transcription levels in both the head kidney and hindgut of the micro-CMC group were significantly increased at 28 dpi (Figure 9F).

In the micro-CMC@LIV group, *cd8α* transcription levels in the spleen and head kidney increased, peaking at 3 dpi and 14 dpi, respectively (Figure 9G–I). In the hindgut of the micro-CMC@LIV group, *cd8α* transcription levels exhibited a biphasic pattern, increasing at 3 dpi and again at 28 dpi (Figure 9G–I). In the LIV group, *cd8α* transcription levels in the spleen remained like the control group, while levels in the head kidney increased at 14 dpi (Figure 9G,H). In the hindgut of the LIV group, *cd8α* transcription levels peaked at 3 dpi and then decreased (Figure 9I).

The transcription levels of *IL-1β* in the spleen, head kidney, and hindgut of the micro-CMC@LIV group increased from 3 to 7 dpi and then decreased. In the LIV group, *IL-1β* transcription increased at 3 and 21 dpi in the spleen, while remaining low in the head kidney and hindgut (Figure 9J–L). In the micro-CMC group, increased *IL-1β* transcription was observed in the head kidney at 28 dpi and hindgut at 21 dpi (Figure 9K,L).

## 4. Discussion

Oral administration has been recognized as a more practical and convenient method for immunization [50,51]. In this study, CMC microcapsules were used as a carrier for oral vaccine delivery to enhance vaccine efficacy. The mean diameter of these microcapsules was 7.37 ± 0.47 μm. Previous studies have confirmed that biocompatible particles with a diameter of less than 10 μm can remain stable in the gastrointestinal environment and carry high concentrations of inclusions [39]. Loading efficiency, which indicates the amount of LIV incorporated into the microcapsules, was determined after microcapsule production [35]. The mean loading efficiency in this study was 88.67 ± 0.36%, consistent with the literature [36].

The midgut and hindgut of fish are considered the primary sites for antigen absorption and have a vital function in immune responses [48,49]; the spleen and kidney are the well-known important immune tissues in fish [46]. The stability of micro-CMC in gastric conditions and the release of encapsulated antigens in the midgut and hindgut are critical for immune activation [38]. In vitro experiments demonstrated that micro-CMC was stable in SGF and released its contents in SIF (Figure 3), similar to other microcapsule systems [37]. The detection of fluorescent micro-CMC@C6 in intestinal content smears from the midgut and hindgut (Figure 4) further confirmed the stability of micro-CMC in gastric conditions and its subsequent entry into the gut. Previous studies on oral vaccination have employed antigen detection in the gut to confirm vaccine delivery [16]. In this study, the detection of MCP protein revealed that at 3 to 9 h post-oral vaccination, viral MCP was present in both the midgut and hindgut of the micro-CMC@LIV group (Figure 5A–F), indicating that the coated antigen was delivered to the midgut and hindgut, where it was released and absorbed by intestinal villi. While the LIV group also showed vaccine delivery to the midgut and hindgut, the antigen signals were transient (Figure 5G–L). This difference may be attributed to increased vaccine degradation in the gastric environment due to the lack of a protective coating.

Neutralizing antibodies play a crucial role in protecting against viral diseases [52]. Yeast surface display technology used for oral vaccination against LMBV achieved a maximum neutralizing antibody titer of 1:85 at 28 dpi and an RPS of 66.66% [18]. Yi et al. developed an LMBV DNA vaccine that induced a maximum antibody titer of 1:375 at 14 dpi and an RPS of 63% [4]. The oral antigen–adjuvant fusion vaccine *P*-MCP-FlaC reduced viral load and achieved an RPS of 62% [53]. In this study, the neutralizing assay demonstrated that the micro-CMC@LIV group elicited higher neutralizing antibody titers (1:81.71) compared to the LIV group (1:21.69). This difference may be attributed to reduced antigen degradation in the gastric environment in the micro-CMC@LIV group. Furthermore, during the LMBRaV challenge, *M. salmoides* in the micro-CMC@LIV group exhibited a higher RPS (82.14%) than the LIV group (61.61%). These results suggest that CMC microcapsules could reduce antigen degradation and enhance protective immunity. Additionally, the lower viral loads in the liver, spleen, and head kidney of the micro-CMC@LIV group compared to the control group and the LIV group indicate that micro-CMC@LIV could stimulate immune responses and reduce viral replication, leading to increased RPS. CMC may be a viable vaccination delivery method for fish. Further research is needed to explore its potential applications in other species.

Antigen recognition and presentation are crucial steps in the adaptive immune response. *MHC II* molecules, expressed on the surface of antigen-presenting cells (APCs), present antigens to CD4+ T helper cells, which are essential for antibody production [54]. In teleost fish, antigen presentation occurs in the head kidney, spleen, and intestine [55,56]. The significant upregulation of *mhc II* transcription levels, particularly in the spleen, head kidney, and intestine of the micro-CMC@LIV group, suggests that the coated vaccine stimulated enhanced antigen presentation, potentially due to reduced antigen degradation in the gastric environment. *IgM*, a major immunoglobulin isotype in teleost fish, is fundamental to systemic immune responses [17]. Upregulation of *igM* transcription was observed in the spleen of both the micro-CMC@LIV and LIV groups. However, in the head kidney, the peak *igM* expression level was observed significantly earlier in the micro-CMC@LIV group (7 dpi) compared to the LIV group (28 dpi) (Figure 9D–F). This accelerated response may contribute to the higher antibody titers observed in the micro-CMC@LIV group. Similarly, upregulation of *mhc II* and *igM* genes has been reported in the head kidney, spleen, and intestine of *M. salmoides* following oral vaccination with yeast-based vaccines [15,17,18]. These findings suggest that the head kidney, spleen, and intestine are important sites for antigen presentation and antibody production after oral vaccination in *M. salmoides*. Notably, upregulation of *igM* was also observed in the intestine of the micro-CMC group, suggesting that CMC may stimulate an *igM* antihapten immune response [57]. T lymphocytes are key players in specific cellular immunity, recognizing antigen fragments presented by *MHC II* molecules [58]. *CD8α*, a surface receptor on CD8+ T cells, serves as the marker of active cytotoxic T cells [59]. In the micro-CMC@LIV group, *cd8α* transcription was significantly upregulated in the head kidney, spleen, and intestine, while no upregulation was detected in the spleen of the LIV group (Figure 9G–I). A previous study revealed that oral vaccination with the LMBRaV *pichia pastoris* vaccine also induced *cd8α* transcription in the gut and head kidney [17]. These results suggest that the coated oral vaccine may induce T-cell activation in multiple tissues. Interleukin-1β is a key cytokine involved in innate immune responses, including the invasion of microbes, damage to tissue, immune responses, and inflammatory responses [60]. Previous studies have shown that *IL-1β* triggers inflammatory responses in the head kidney, spleen, and intestine following immunization with LMBV DNA and recombinant oral vaccines [4,18]. In this study, *IL-1β* transcription increased rapidly and peaked at 7 dpi in the head kidney, spleen, and intestine of the micro-CMC@LIV group, while it was downregulated in the intestine and head kidney of the LIV group (Figure 9J–L). These findings indicate that the coated vaccine induced inflammatory responses in multiple tissues. Overall, these results suggest that micro-CMC can effectively deliver antigens to the intestine and stimulate immune responses, both adaptive and innate, across multiple tissues of *M. salmoides*.

## 5. Conclusions

This study demonstrated that microcapsules can effectively protect and deliver antigens within the gastrointestinal tract of *M. salmoides*. The micro-CMC@LIV vaccine significantly stimulated both innate and adaptive immune responses against LMBRaV in *M. salmoides*, resulting in higher RPS and lower viral loads. These findings suggest that micro-CMC@LIV has the potential to be developed into a vaccine candidate and could serve as an effective method for preventing LMBRaV disease in *M. salmoides*.

## Figures and Tables

**Table 1:** Primers used in this study.

Name	Sequence (5′–3′)	Usage	Accession No. or Reference
*mcp*	F-CCCGTGGGTTGGTTTACCAA-R R-CCGCCAGCAGTTTAATCTGG-F	dd PCR	GU256635
*mhc II*	F-GGGATGGAGACCAGGCGATA-R R-CCCGCTTGACAGCACATCCT-F	qRT-PCR	XM_038735684
*igM*	F-CGACTACGATATGAACTGGG-R R-GCTGTTGTCTCTGGAGATGG-F	qRT-PCR	XM_038698199
*cd8α*	F-CCAAGTCAGTGCACATCTAC-R R-GGGCCCAGTATGATTGAAGG-F	qRT-PCR	XM_038696403
*IL-1β*	F-CCGTGCCAACAGTGTGAAGA-R R-GGGTGCTGTGTCCACCTTGC-F	qRT-PCR	XM_038733429
*β-actin*	F-CCACCACAGCCGAGAGGGAA-R R-TCATGGTGGATGGGGCCAGG-F	qRT-PCR	[45]

## Data Availability

The datasets generated for this study are available upon request to the corresponding author.

## Supplementary Materials

The following supporting information can be downloaded at: https://www.mdpi.com/article/10.3390/vaccines13030233/s1, Figure S1: Histopathological observation of largemouth bass post micro-CMC treatment; Figure S2: Histopathological observation of largemouth bass post LIV oral administration; Figure S3: Safety Evaluation of the LMBRaV inactivated vaccine; Figure S4: Immunofluorescence analysis of the midgut and hindgut sections of the micro-CMC@LIV group at 6 h post-vaccination without anti-LMBRaV MCP polyclonal antibody (A,C), and the midgut and hindgut sections of the control group (B,D). Scale bar = 100 µm.
