# Supplementary material for: The Protective Effects and Immunological Responses Induced by a Carboxymethyl Cellulose Microcapsule-Coated Inactivated Vaccine Against Largemouth Bass Ranavirus (LMBRaV) in Largemouth Bass (Micropterus salmoides)
Source: Vaccines (Basel). 2025 Feb 25;13(3):233. doi: 10.3390/vaccines13030233 (PMC11946483; doi:10.3390/vaccines13030233)
Supplement: Supplementary file 1 [file vaccines-13-00233-s001.zip › vaccines-3442651-supplementary.pdf]

Supplementary Figures

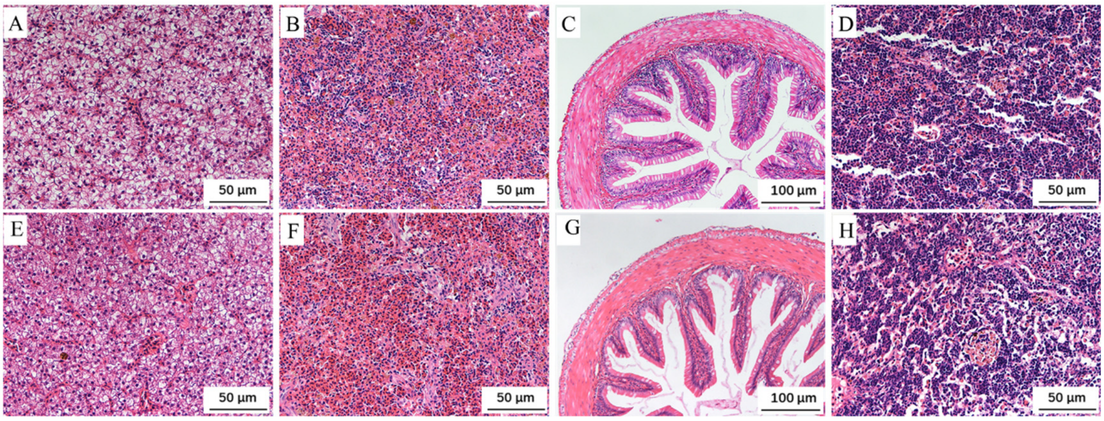

Figure S1. Histopathological observation of largemouth bass post micro-CMC treatment. A-D: The liver, spleen, intestine and head kidney of the control group fish; E-H: the liver, spleen, intestine and kidney of the micro-CMC group fish. Scale bar = 50 µm (A, B, D, E, F, H), 100 µm (C, G).

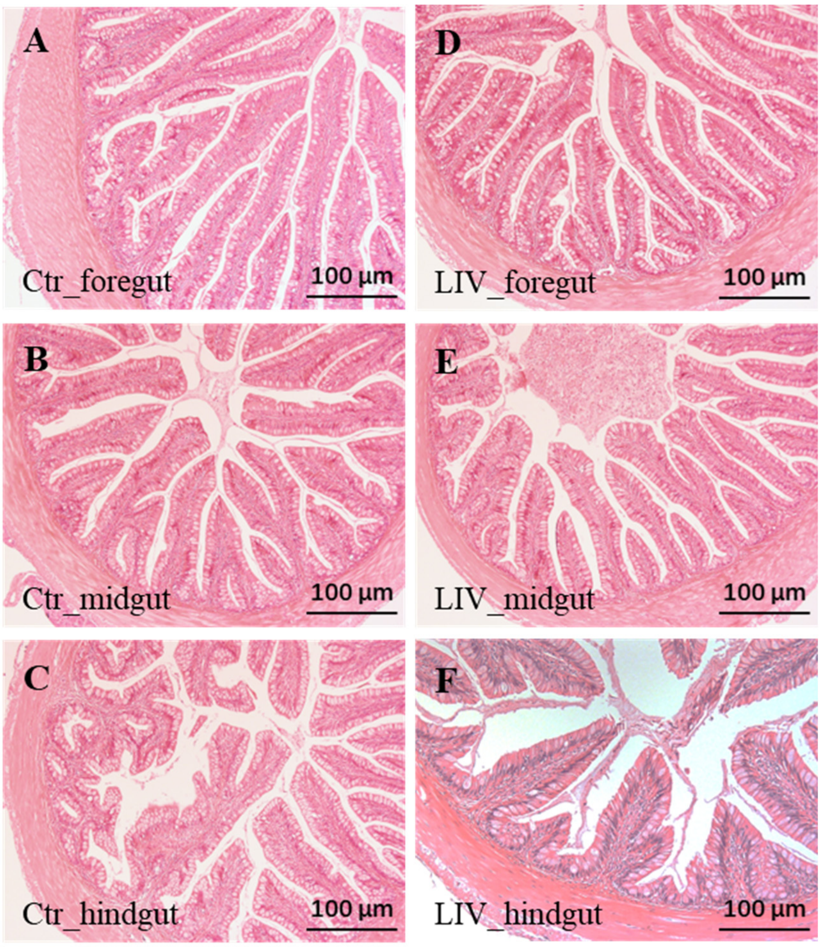

Figure S2. Histopathological observation of largemouth bass post LIV oral administration. A-C: The foregut, midgut and hindgut of the control group fish; D-F: the foregut, midgut and hindgut of the LIV group fish. Scale bar = 100 µm.

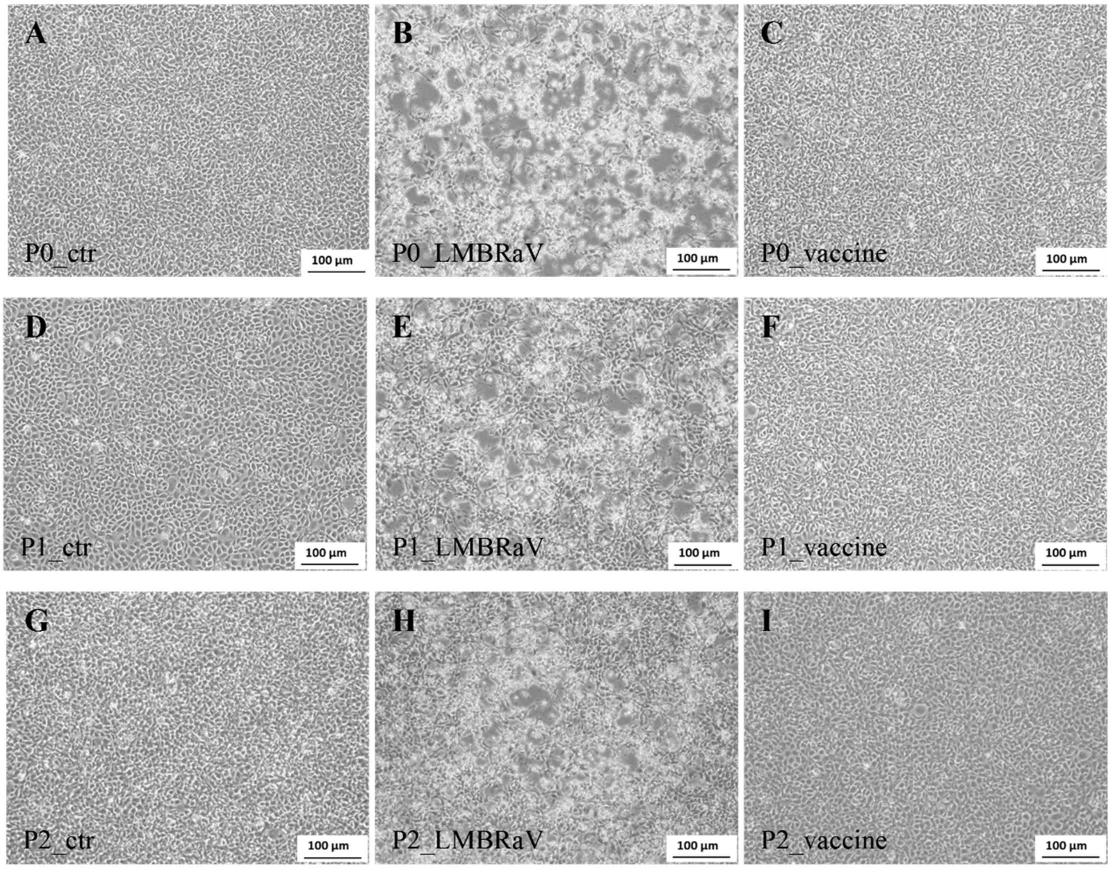

Figure S3. Safety Evaluation of the LMBRaV inactivated vaccine. (A, D, G) Mock-infected EPC cells. EPC cells were initially infected (passage 0 [P0]) with LMBRaV at 72 hours post-infection (hpi) (B); the P0 supernatant was used to infect cells for a second passage (P1) at 72 hpi (E); and the P1 supernatant was used to infect cells for a third passage (P2) at 72 hpi (H). EPC cells initially infected (P0) with the vaccine at 72 hpi (C); the P0 supernatant was used to infect cells for a second passage (P1) at 72 hpi (F); and the P1 supernatant was used to infect cells for a third passage (P2) at 72 hpi (I). Scale bar = 100 µm

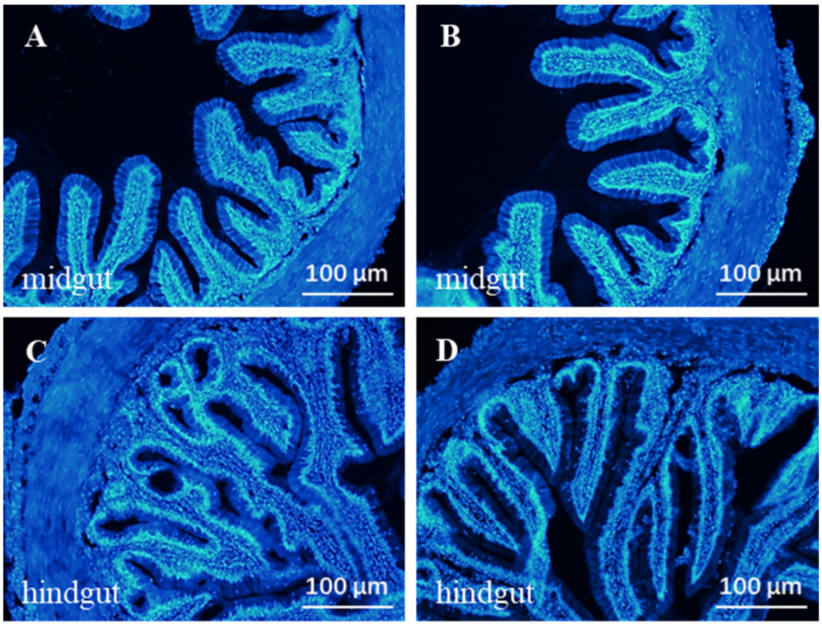

Figure S4. Immunofluorescence analysis of the midgut and hindgut sections of the micro-CMC@LIV group at 6 h post-vaccination without anti-LMBRaV MCP polyclonal antibody (A, C), and the midgut and hindgut sections of the control group (B, D). Scale bar = 100 μm.
